# Stability, enrichment, and quantification of total and HPV16-specific IgG present in first-void urine

**DOI:** 10.1038/s41598-024-65257-0

**Published:** 2024-06-23

**Authors:** Laura Téblick, Marijana Lipovac, Margo Bell, Annemie De Smet, Ingrid De Meester, Peter Delputte, Alex Vorsters

**Affiliations:** 1 Centre for the Evaluation of Vaccination (CEV), Vaccine & Infectious Disease Institute (VAXINFECTIO), Faculty of Medicine and Health Sciences, University of Antwerp, 2610 Wilrijk-Antwerp, Belgium; 2 Laboratory of Medical Biochemistry, Faculty of Pharmaceutical, Biomedical and Veterinary Sciences, University of Antwerp, 2610 Wilrijk-Antwerp, Belgium; 3 Laboratory for Microbiology, Parasitology and Hygiene (LMPH), Faculty of Pharmaceutical, Biomedical and Veterinary Sciences, University of Antwerp, 2610 Wilrijk-Antwerp, Belgium

**Keywords:** Immunology, Infectious diseases

## Abstract

First-void urine (FVU) samples, containing human papillomavirus (HPV)-specific IgG from female genital tract secretions, provide a non-invasive option for disease monitoring and vaccine impact assessment. This study explores the utility of FVU for IgG quantification, exploring stability and compatibility with DNA preservation methods, alongside various IgG enrichment methods. Healthy female volunteers provided FVU and serum samples. FVU was collected with or without urine conservation medium (UCM) and stored under different conditions before freezing at −80 °C. Four IgG enrichment methods were tested on FVU samples. All samples were analyzed using three total human IgG quantification assays and an in-house HPV16-specific IgG assay. Samples stored with UCM buffer had higher total and HPV16-specific IgG concentrations (p ≤ 0.01) and IgG remained stable for at least 14 days at room temperature. Among IgG enrichment methods, Amicon filtration (AM) and AM combined with Melon Gel purification (AM-MG) provided similar HPV16-IgG concentrations, correlating strongly with serum levels. Protein G magnetic beads methods were incompatible with time-resolved fluorescence-based assays. This study highlights FVU as a reliable and convenient sample for IgG quantification, demonstrating stability for at least 14 days at room temperature and compatibility with UCM DNA preservation. It emphasizes the need to select appropriate IgG enrichment methods and confirms the suitability of both AM and AM-MG methods, with a slightly better performance for AM-MG.

## Introduction

First-void urine (FVU) samples have emerged as a valuable source for non-invasively monitoring human papillomavirus (HPV) vaccination and related disease, as it contains immunological, virological, and diagnostic information, including HPV-specific immunoglobulins (Ig), HPV DNA, and methylation markers^1–10^. Collecting this biological information through FVU sampling offers distinct advantages over invasive methods such as blood collection and cervical smears, as it allows for convenient self-collection at home^11,12^.

FVU is characterized by its rich concentration of DNA, proteins, bacteria, viruses, and other female genital tract (FGT) secretions exceeding those found in mid-stream urine^8^. This results in elevated levels of biomarkers of interest although it does introduce greater complexity, which can potentially challenge the stability, isolation/extraction, and quantification of these biomarkers^2,13^. To address one of these issues, the use of a DNA stabilization buffer has become a standard practice to preserve the integrity of nucleic acids in FVU samples^2,14,15^. However, the impact of such buffers on the stability and integrity of IgG in FVU, has not been explored. Furthermore, while studies have demonstrated urinary IgG stability under various conditions for specific viral pathogens^16,17^, a knowledge gap exists regarding the stability of (HPV-specific) IgG in FVU.

Earlier studies showed that FVU contains limited quantities of IgG, approximately 0.5% of total IgG and 0.07% of HPV-specific IgG compared to serum levels^18^. While these studies have demonstrated good correlations between serum and FVU HPV-specific antibodies, FVU samples still exhibit lower antibody positivity than serum^5,6,18^. Apart from assay optimization, there lies potential in enhancing FVU sample preprocessing to increase antibody concentration, thus enabling the detection of even lower antibody titers in FVU. Additionally, inter- and intra-individual variation has been reported in FGT secretions^19^, underlining the importance of normalizing HPV-specific antibody concentrations, where total human IgG could play a crucial role. To address these aspects, this study explores various IgG enrichment and quantification methods that have never been evaluated on FVU samples.

This study is the first to evaluate the stability of IgG in FVU, assess its compatibility with a commonly used FVU DNA stabilization buffer, and compare diverse approaches for enriching and quantifying IgG. The outcomes of this study serve as fundamental steps in demonstrating the suitability of this sample type not only for disease monitoring but also for assessing vaccine impact, ultimately having broad implications for clinical diagnostics and public health interventions^20^.

## Materials and methods

### Study population

Women, aged between 20 and 50 years old, employed at the University of Antwerp were recruited by addressing them personally. Next, the study team briefly informed the participants and provided them with the information brochure, including an informed consent form. They were asked to read these documents thoroughly before participating in the study. Informed consent was obtained from all volunteers, and data and samples were coded to ensure privacy of the participants. Key exclusion criteria included individuals who had previously received treatment for cervical cancer or precancerous lesions. This study was approved by the Institutional Review Board of UZA/ University of Antwerp (B300201734129).

### Sample collection and storage

For each analysis, women were asked to provide FVU samples. Before collecting a FVU sample, the participants were instructed not to wash their genitals thoroughly, not to use a tampon, and to avoid urinating for at least two hours before collection. FVU samples were collected using the Colli-Pee^®^ 20 ml device (Novosanis, Belgium).

**Figure 1:**
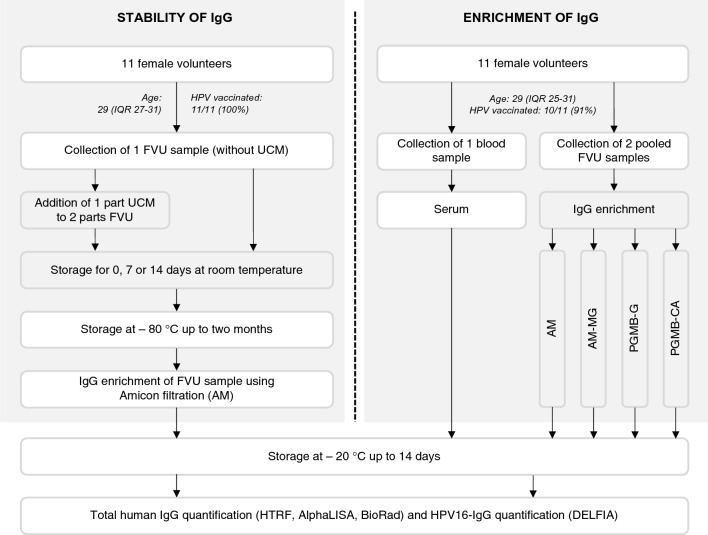
Study design. To assess storage and stability of IgG in FVU, eleven women provided a FVU sample collected without UCM. To 10 ml of the FVU sample, UCM was added in a 1:2 UCM:FVU ratio. The samples were aliquoted in 4 ml samples and stored at RT for different durations whereafter they were stored at – 80 °C. All samples were processed using Amicon filtration. To evaluate different IgG enrichment methods, eleven women provided both blood samples and two pooled FVU samples. From the blood samples, serum was extracted, while the pooled FVU samples were aliquoted in 4 ml samples and processed using different protocols. The different IgG enrichment protocols were: Amicon filtration (AM), AM followed by melon gel purification (AM-MG), protein G magnetic bead purification using glycine elution (PGMB-G) and citric acid elution (PGMB-CA). All samples were analyzed for total human IgG using the HTRF and AlphaLISA technology from PerkinElmer, as well as the BioPlex isotyping assay from Bio-Rad. Additionally, HPV16-IgG concentrations were measured using the HPV16 DELFIA.

To assess IgG stability and compatibility of IgG storage with urine conservation medium (UCM) (Novosanis, Belgium), 11 female volunteers collected one FVU sample without UCM (Fig. 1). Ten ml FVU was transferred to a Falcon tube and 5 ml UCM was added to obtain a UCM:FVU ratio of 1:2. All samples were vortexed and aliquoted in 4 ml aliquots for UCM buffered samples and in 2.66 ml aliquots for samples without UCM. A UCM buffered and unbuffered aliquot was 1) directly stored at − 80 °C, 2) stored at room temperature (RT) for seven days before storage at − 80 °C, 3) stored at RT for 14 days before storage at − 80 °C. The remaining aliquots were stored immediately at − 80 °C for future use.

To compare different FVU enrichment methods, again 11 volunteers—partially overlapping with those from the stability analysis, collected two FVU samples using collection devices prefilled with 1/3 UCM with a time interval of at least two hours between two collections (Fig. 1). The two samples were pooled in a 50 ml Falcon tube to reach a final volume of approximately 26.7 ml FVU and 13.3 ml UCM. Pooled samples were vortexed and aliquoted in 4 ml aliquots. All aliquots were stored immediately at − 80 °C before processing. We measured the pH of all samples and determined the presence of erythrocytes using Hemastix^®^ reagent strips (Siemens Healthcare Diagnostics Inc., Dilbeek, Belgium). Paired blood samples were collected using 10 ml BD Vacutainer^®^ Serum Tubes without anticoagulant (BD Benelux N.V., Erembodegem, Belgium). The blood samples were allowed to clot for 30–60 min, whereafter they were centrifuged for 10 min at 1000 × g and 20 °C. The serum was divided into aliquots before storage at – 80 °C. All aliquots were registered in the Antwerp Biobank (Biobank Antwerpen, Antwerp, Belgium; ID: BE71030031000) before further analysis.

### Sample processing

To compare different enrichment methods, FVU samples were subjected to four different protocols. The first method included centrifugation of a 4 ml FVU aliquot at 4000 × g for 20 min at 20 °C in an Amicon Ultra-4 50 K filter device (Merck Millipore, Belgium), this method is further referred to as AM. Centrifugation continued until the volume of FVU remaining on the filter was less than 50 µl. Next, the sample was diluted with dPBS until a total volume of 500 µl was reached. The second method included AM filtration and an extra purification step using the Melon^™^ Gel IgG Spin Purification Kit (Thermo Scientific, Belgium), this method is further referred to as AM-MG. Here, a 4 ml FVU aliquot was centrifuged using the Amicon Ultra-4 50 K filter device as mentioned above. The sample was diluted with 450 µl purification buffer to obtain a final 1:10 dilution of the sample and further purified according to the manufacturer’s instructions. All centrifugation steps were performed at 4000 × *g*. For the next two methods, Pierce^™^ Protein G magnetic beads (PGMB) were used (Thermo Scientific, Belgium). For each 4 ml FVU aliquot, 0.5 mg PGMB were placed into two 2 ml Protein Lo-Binding microcentrifuge tubes (1 mg PGMB per sample in total). According to the manufacturer’s instructions, the beads were washed twice using binding-wash buffer (0.05 M Tris, 0.15 M NaCl, 0.05% Tween 20) and a DynaMag^™^-2 Magnet (Invitrogen^™^, Belgium). Afterwards, the 4 ml FVU aliquots were added to the beads by adding 2 ml in each prepared tube. Tween detergent was added to a final concentration of 0.05%. The samples were incubated for approximately one hour at RT with end-over-end mixing. Magnetic beads were collected with the magnet, and the supernatant was discarded. 500 µl binding-wash buffer was added to the first tube, the beads were dissolved, and the dissolved beads were added to the same sample’s second tube. The beads were washed three times whereafter the total IgG was eluted in 100 µl using two different elution buffers; 0.1 M glycine at pH 2 (further referred to as PGMB-G), and 0.1 M citric acid (further referred to as PGMB-CA) at pH 2.5. Four additional elution buffers were tested: 1) 0.1 M glycine elution at pH 3.5, 2) 0.1 M citric acid elution at pH 3.5, 3) elution using the Pierce™ Gentle Ag/Ab Elution Buffer (Thermo Fisher Scientific, Dilbeek, Belgium), and 4) elution using the Pierce™ IgG Elution Buffer (Thermo Fisher Scientific, Dilbeek, Belgium). After elution, the samples were neutralized using 1 M Tris at pH 8.5. All samples were brought to a pH of 7–7.5 using NaOH or HCl. For the storage and stability analysis, all aliquots were processed using the Amicon Ultra-4 50 K filter device as mentioned above. The concentrate was diluted in PBS until a final volume of 500 µl was reached. All purified samples were stored up to 14 days at − 20 °C before further analysis. No purification step was performed for serum samples.

### Total human IgG quantification

#### Bio-Rad BioPlex luminex assay

Total human IgG concentrations were assessed for both processed FVU samples and for serum samples using the BioPlex Pro^™^ Human Isotyping Assay for total human IgG (Bio-Rad, USA) following the manufacturer’s instructions. For FVU samples, dilutions of 1:128 and 1:512 were used, while serum samples were diluted at 1:10,000 and 1:100,000. UCM, diluted similarly to the samples was used as control. The LX200 platform (Luminex, Austin, Texas, USA) was used for measurement, and concentrations were derived from median fluorescence intensity (MFI) values using a five-parameter logistic regression. The average of two dilution-corrected concentrations was reported as the total human IgG concentrations. The dynamic range of this assay is 3.00–30,270 ng/ml.

#### HTRF homogeneous assay

The second total IgG quantification method used was the homogeneous time-resolved fluorescence (HTRF) Cisbio Human IgG kit, designed for the fast quantification of human IgG (Cisbio, Belgium). After optimization experiments, FVU dilution 1:300 and serum dilution 1:500,000 were selected for total human IgG quantification to fit within the assay’s dynamic range. Other dilutions tested were 1:100, 1:1000, and 1:10,000 for FVU and 1:1000, 1:10,000, 1:100,000, and 1:1,000,000 for serum. UCM, diluted similarly to the samples, was used as control. Total IgG concentrations were determined according to the manufacturer’s instructions, and signals were measured for 665 nm and 620 nm using the Victor Nivo multimode plate reader (Perkin Elmer, Belgium). The measurement ratio (665 nm signal/ 620 nm signal) × 10^4^ was calculated for each measurement and the average background well signal was subtracted from all values. Total human IgG concentrations were calculated using the sigmoidal dose–response curve with variable slope. All calculations were performed using GraphPad Prism version 9.5.1. The dynamic range of this assay is 0.91–2,000 ng/ml.

#### AlphaLISA assay

The third total human IgG immunoassay used was the homogeneous highly sensitive IgG (human) Amplified Luminescent Proximity Homogeneous Assay (AlphaLISA) Detection Kit (Perkin Elmer, Belgium) for total IgG quantification. Similar to the previous assays, optimized dilutions of 1:300 for FVU and 1:500,000 for serum samples were applied to fit within the assay’s dynamic range. UCM, diluted similarly to the samples, was used as control. The kit was used according to the manufacturer’s instructions. A sharp peak of light emission at 615 nm was measured using the Alpha technology on the Victor Nivo multimode plate reader (Perkin Elmer, Belgium). The average signal of four background wells was subtracted from all sample values before analysis. Concentrations were calculated using nonlinear regression with the 4-parameter logistic equation. A sigmoidal dose–response curve with variable slope was constructed using 1/Y^2^ data weighting. All calculations were performed using GraphPad Prism version 9.5.1. The dynamic range of this assay is 0.24–1000 ng/ml.

### HPV16-IgG quantification (DELFIA)

HPV16-specific IgG were measured for all processed FVU samples and serum samples using an in-house Dissociation-Enhanced Lanthanide Fluorescent immunoassay (DELFIA). Sample testing was performed using the HPV DELFIA protocol as described previously^21^. For FVU samples, the testing began undiluted, while serum samples, standards, and controls were started at a dilution of 1:400. Serial dilutions at a 1:2 ratio were made for each sample, and four dilutions were tested. Plates were read using either the EnVision^®^ multimode plate reader (Perkin Elmer, Belgium) or the Victor Nivo^®^ multimode plate reader (Perkin Elmer, Belgium). Background counts were subtracted from the sample measurements. To determine IgG concentrations, the parallel line method (PLL) described in the World Health Organization (WHO) HPV Labnet Manual 2009 was employed^22^. Samples were assigned titers if they exhibited linear titration and fulfilled all PLL conditions. The results are reported as HPV16 IgG titers in International Units (IU/ml). The HPV16 (05–134) standard (10–140) used in the assay was obtained from the National Institute for Biological Standards and Controls [NIBSC] (Potter’s Bar, UK). The results of the HPV16-DELFIA assay were in-house compared to the concentrations derived with the M9ELISA^5,23^, and excellent Spearman rank correlations were established (≥ 0.9) (data not shown).

### Statistical analysis

R statistical software version 4.2.2 was used for statistical analysis. Firstly, normality of the data was assessed using the Shapiro–Wilk test, assuming that the data on one variable were monotonically related to the other variable. If the data followed a normal distribution, significant differences between different storage conditions and IgG enrichment methods were analyzed using paired t-tests. However, if the data did not meet the normality assumption, non-parametric Wilcoxon signed-rank testing was applied. Statistical significance was determined as p-adjusted < 0.05, and the Holm-Bonferroni method was used for adjusting p-values to counteract the problem of multiple comparisons. To explore potential correlations between human IgG quantification kits and between paired FVU and serum, Spearman’s rank correlations were calculated. Additionally, we generated Bland Altman plots to assess concordance between the total IgG quantification kits^24^. To evaluate the precision of these correlations, bootstrap 95% confidence intervals (CI) were estimated through 1,000 replicates.

### Ethics approval

The study was conducted according to the guidelines of the Declaration of Helsinki and approved by the Institutional Review Board (or Ethics Committee) of UZA/University of Antwerp (B300201734129).

## Results

### Population and sample characteristics

For stability and UCM compatibility analyses, 11 healthy female volunteers with a median age of 29 (IQR 27-31) provided two FVU samples, and 100% (11/11) reported being fully vaccinated with one of the HPV vaccines (Fig. 1). Similarly, to compare IgG enrichment methods for FVU, 11 healthy female volunteers with a median age of 29 (IQR 25-31) provided paired FVU and serum samples, with 91% (10/11) of the participants reporting being vaccinated against HPV (Fig. 1). Notably, 6 out of the 11 participants provided samples for both analyses. All participants reported to not have had an HPV infection in the previous six months and the erythrocyte count in all samples was negative.

### IgG storage and stability in FVU

**Figure 2:**
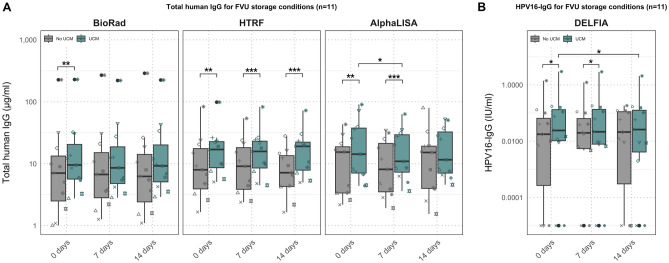
Total IgG and HPV16-IgG results for different storage conditions of FVU. (**A**) Boxplots of total human IgG concentrations for 11 samples stored under different conditions using the BioPlex, the HTRF assay and the AlphaLISA. (**B**) Boxplots of HPV16-IgG concentrations. Significance levels are represented in the figure by an asterisk (*p < 0.05, **p < 0.01, ***p < 0.001). Outliers in all figures represent the same ID.

We assessed the stability of IgG in FVU under various storage conditions and evaluated the compatibility of the UCM buffer with IgG storage. Samples were stored with or without UCM buffer at various durations at RT before freezing at − 80 °C. Three different quantification methods were compared to measure total human IgG concentrations, and HPV16-IgG levels were measured using the HPV16 DELFIA (Supplementary Table 1, Fig. 2).

Using the BioRad assay, total human IgG concentrations were significantly higher in samples stored with the UCM buffer than those without when samples were immediately stored at − 80 °C (p = 0.005). For the HTRF assay, significantly higher total IgG concentrations were quantified for samples stored with UCM buffer for all RT storage durations (p ≤ 0.002). The AlphaLISA assay showed significantly lower IgG concentrations for samples stored with UCM (14.26 µg/ml) than those without UCM (15.37 µg/ml) for those directly stored at − 80 °C (p = 0.005) and significantly higher (10.92 µg/ml vs. 8.11 µg/ml) for samples stored at RT for 7 days (p = 0.001). Furthermore, significantly higher total human IgG concentrations were quantified with the AlphaLISA for UCM buffered samples directly stored at − 80 °C (14.26 µg/ml) compared to those stored at RT for 7 days before storage at − 80 °C (10.92 µg/ml) (p = 0.02) (Fig. 2A). The HPV16-IgG concentrations were significantly higher in samples stored with UCM buffer compared to those without UCM buffer, both for samples directly stored at − 80 °C (p = 0.02) and those stored at RT for 7 days before storage at − 80 °C (p = 0.04). Additionally, a significantly lower median HPV16-IgG concentration was observed for UCM buffered samples directly stored at − 80 °C (0.024 IU/ml) compared to those stored at RT for 14 days before storage at − 80 °C (0.026 IU/ml) (p = 0.02) (Fig. 2B). The UCM control wells did not provide signals above the LOD for each total IgG measurement method.

**Figure 3:**
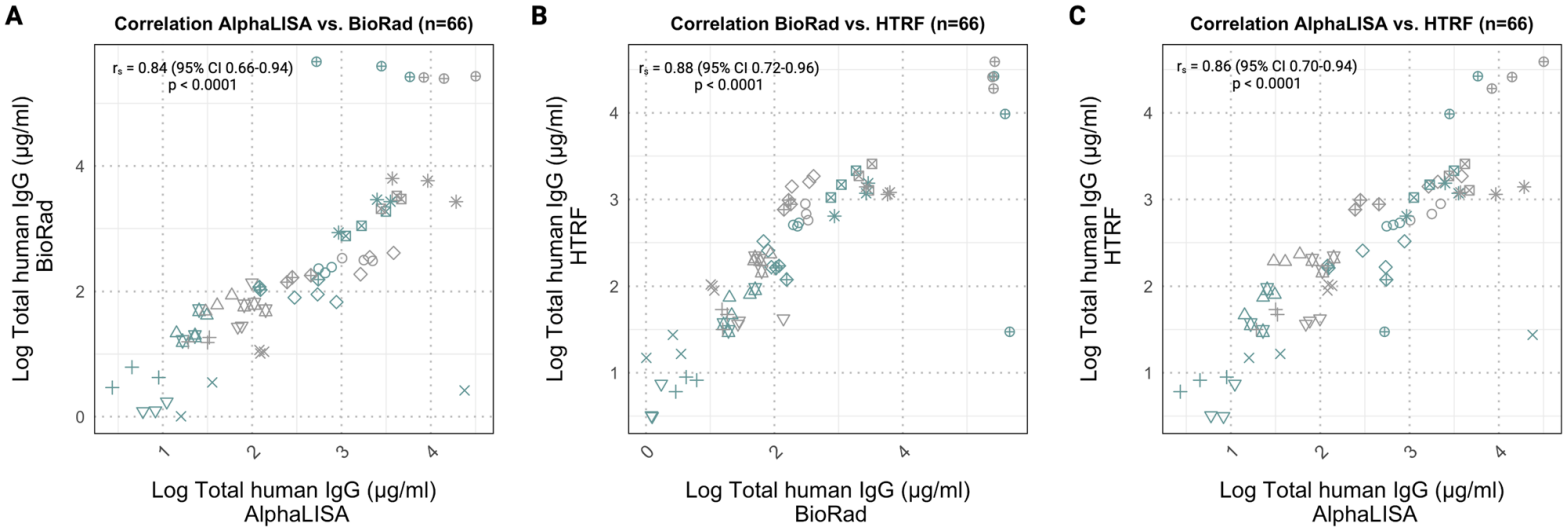
Spearman correlation plots for all stability data combined (**A**) Correlation plot between the AlphaLISA and the BioRad assay. (**B**) Correlation plot between the BioRad assay and the HTRF assay. (**C**) Correlation plot between the AlphaLISA and the HTRF assay. Spearman’s rank correlation coefficient presented in the figure are for all conditions combined. Different symbols represent IDs and different colors represent buffer conditions in the correlation plots. Outliers in all figures represent the same ID.

Significant Spearman rank correlations were found among the three total human IgG immunoassays, with overall coefficients of 0.84 (95% CI 0.66-0.94) or higher (Fig. 3). The highest correlation coefficient was observed between BioRad and HTRF (Fig. 3B). Additionally, Bland Altman plots indicate that largest absolute differences between total human IgG methods are found for samples with higher total human IgG concentrations (Supplementary Fig. 1).

### IgG enrichment in FVU

**Figure 4:**
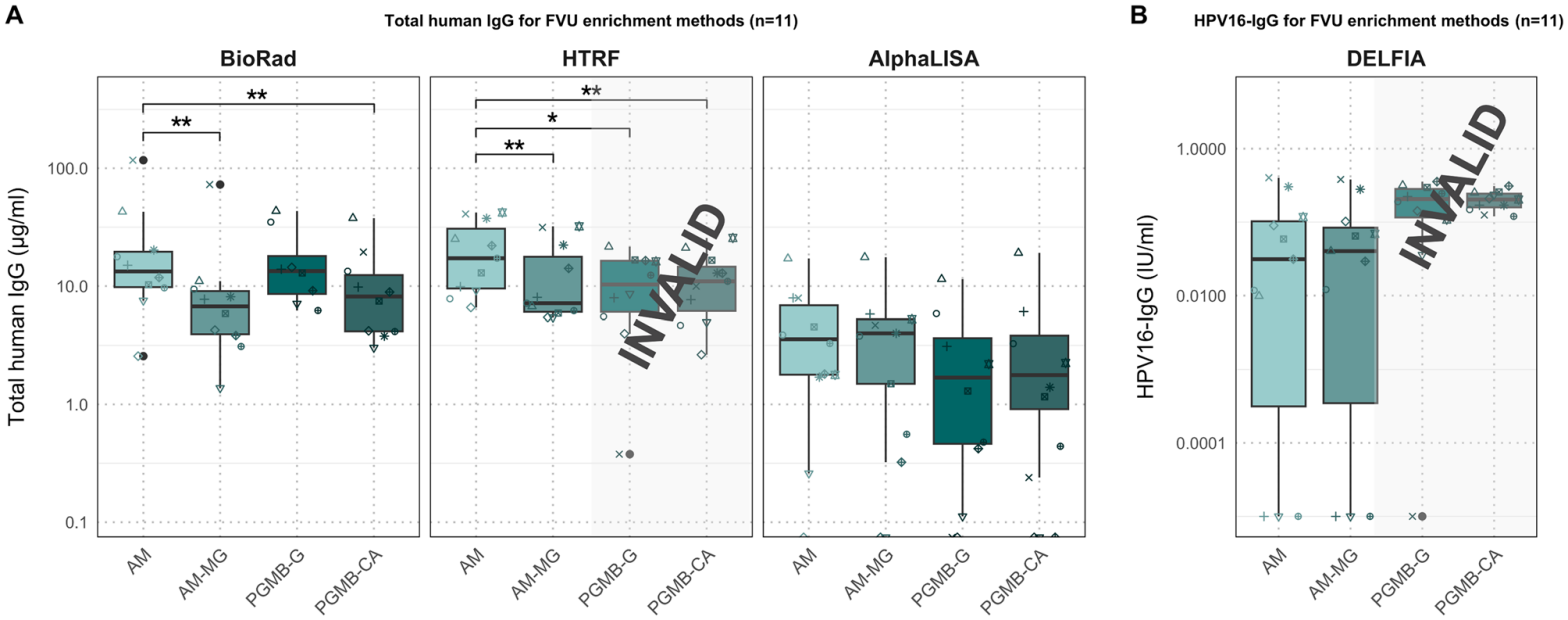
Total IgG and HPV16-IgG results in FVU of the IgG enrichment cohort. (**A**) Boxplots of total human IgG concentrations after different enrichment protocols for 11 FVU samples using the BioPlex, the HTRF assay and the AlphaLISA. (**B**) Boxplots of HPV16-IgG concentrations after different enrichment protocols for 11 FVU samples. Significance levels are represented in the figure by an asterisk (*p < 0.05, **p < 0.01, ***p < 0.001). Different symbols represent different IDs. Results for TRF based methods (HTRF and DELFIA) are invalid for PGMB enrichment methods due to non-specific binding.

We evaluated four different methods for enriching IgG in FVU samples. All processed samples were then analyzed using three distinct total human IgG kits, and we used the DELFIA assay for HPV16-IgG measurement (Fig. 1, Supplementary Table 2). Results for PGMB-CA and PGMB-G enrichment methods tested using TRF based methods were reported as invalid due to non-specific binding. For the BioRad total IgG assay, significantly higher concentrations were found for AM compared to AM-MG (p = 0.004) and PGMB-CA (p = 0.004) (Fig. 4). The HTRF kit provided significantly higher total IgG concentrations for AM compared to AM-MG (p = 0.001), PGMB-G (p = 0.005), and PGMB-CA (p = 0.03). However, no significant differences were observed between the enrichment methods for the AlphaLISA (p ≥ 0.06) (Fig. 4A). For the AlphaLISA, samples processed with the PGMB methods gave signals close to the LOQ but above LOD. No significant differences in HPV16-IgG concentrations were observed between the IgG enrichment methods (p ≥ 0.06) (Fig. 4B).

**Figure 5:**
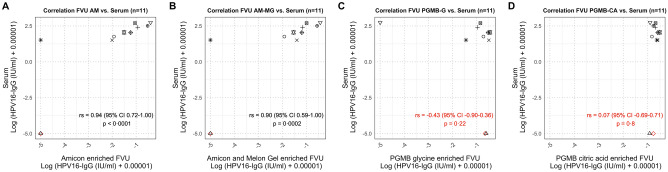
Correlation plots for HPV16-IgG for differently processed FVU samples and serum samples. Note that the results of PGMB-G and PGMB-CA should be interpreted with caution due non-specific binding and insignificant correlations with serum. Different shapes represent different IDs. Vaccinated IDs are presented in black, unvaccinated in red.

For the unvaccinated volunteer, no HPV16-IgG was detected in the serum, nor in the AM and AM-MG purified FVU samples (Fig. 5). Nevertheless, employing the PGMB methods, an HPV16-IgG titer above the median titer was assigned for the unvaccinated volunteer. The Spearman rank correlations between enrichment methods were generally most significant when comparing AM to AM-MG for all quantification methods (p ≤ 0.001), except for AlphaLISA, where the most significant correlation was observed between PGMB-G and PGMB-CA (p ≤ 0.0002) (Supplementary Table 3).

Significant correlation coefficients were found for AlphaLISA vs. BioRad (0.67 (95% CI 0.02–0.96)) and BioRad vs. HTRF (0.72 (95% CI 0.08–0.99)) for AM enriched samples. AM-MG enriched samples showed significant correlations for total IgG AlphaLISA vs. BioRad (0.83 (95% CI 0.37–0.98)) and AlphaLISA vs. HTRF (0.61 (95% CI − 0.03–0.97)). However, no significant correlations were observed between the total IgG quantification kits for the PGMB-G and PGMB-CA enriched samples (p ≥ 0.06) (Table 1). Additionally, Bland Altman plots again indicate that largest absolute differences between total human IgG methods are found for samples with higher total human IgG concentrations (Supplementary Fig. 2).

**Table 1:** Correlation coefficients for the different total human IgG quantification methods for different FVU IgG enrichment methods and serum.

	BioRad vs. HTRF		AlphaLISA vs. BioRad		AlphaLISA vs. HTRF	
	Spearman correlation (95% CI)	Adjusted p-value	Spearman correlation (95% CI)	Adjusted p-value	Spearman correlation (95% CI)	Adjusted p-value
FVU						
AM	0.72 (0.08–0.99)	0.0242	0.67 (0.02–0.96)	0.0394	0.23 (− 0.53–0.81)	0.5031
AM-MG	0.61 (− 0.08–0.97)	0.0802	0.83 (0.37–0.98)	0.0032	0.61 (− 0.03–0.97)	0.04817
PGMB-G	0.50 (− 0.40–0.96)	0.5517	0.65 (-0.16–1.00)	0.0568	0.49 (− 0.38–0.94)	0.1425
PGMB-CA	0.38 (− 0.27–0.90)	0.775	0.51 (-0.27–0.91)	0.1327	0.38 (− 0.36–0.91)	0.2419
Serum	0.26 (− 0.77–0.89)	0.4697	0.04 (-0.74–0.86)	0.9186	− 0.14 (− 0.69–0.65)	0.6935

Additionally, the correlations between the processed FVU samples and the serum samples for HPV16-IgG were examined (Fig. 5). Highly significant Spearman correlation coefficients were observed between HPV16-IgG levels for AM-processed FVU and serum (0.94 (95% CI 0.72–1.00)) and between AM-MG processed FVU and serum (0.90 (95% CI 0.59–1.00)), whereas no significant correlations were found between PGMB-G or PGMB-CA processed FVU, and serum (p ≥ 0.2). For the serum samples, no significant Spearman correlations were found for the three total human IgG kits (p ≥ 0.5) (Table 1) and Bland Altman plots also show poor concordance between the kits (Supplementary Fig. 3).

### Non-working IgG enrichment methods

In addition to the four described IgG enrichment methods, four other elution steps were explored for the PGMB as described in the "Methods" section (data not shown). However, despite these efforts, the samples did not yield quantifiable concentrations using any of the described immunoassays, leading us to exclude these methods from further evaluation.

## Discussion

### FVU as a valuable sample for vaccine and disease monitoring

It is well-established that IgG can be detected in urine samples, serving as a valuable tool for identifying specific IgG related to various infections and diseases^25–29^. FVU has the unique capability of capturing and concentrating FGT secretions, making it an ideal sample for assessing pathogen-specific IgG associated with the FGT^8,9^. Several promising studies have already been performed within the field of HPV DNA detection in FVU^14,30,31^, but certain questions regarding HPV-related immunological information in this sample type remain largely unanswered.

In this study, we aimed to assess the stability of total human and HPV16-specific IgG in FVU, evaluate its compatibility with DNA preservation methods, and explore various methods for enriching and quantifying IgG. Our findings represent a significant step in exploring the potential of FVU as a non-invasive sample for monitoring HPV vaccine trials and epidemiological studies^18^.

### Impact of UCM buffer on IgG stability in FVU

Previous studies focusing on HPV DNA detection in FVU have demonstrated the effectiveness of the UCM buffer in preserving DNA^2,32,33^. To streamline non-invasive sampling for both follow-up of vaccination and infection, we investigated the influence of the UCM buffer, a PBS-based solution containing a chelating agent, a microbicide, a fungicide, and BSA, on IgG stability in FVU. Overall, our findings suggest that the UCM buffer positively affects the detection of total human IgG and HPV16-specific IgG. Only for one assay, the AlphaLISA, we did observe some variating effects of the UCM buffer on the IgG detection. BSA in the UCM buffer likely reduces IgG adhesion to tube surfaces, leading to detection of higher (HPV16-)IgG concentrations^2,34^. Moreover, we observed that IgG remains stable at RT for at least 14 days, with minimal alterations in total and HPV16-specific IgG concentrations over time, even after a freeze–thaw cycle. These findings are particularly valuable in settings where immediate refrigeration or freezing of samples is not feasible.

### Comparison of IgG enrichment methods for FVU

Although HPV-specific antibodies have shown to be adequately detectable in FVU samples enriched using Amicon filtration, lower HPV-antibody positivity rates have been reported compared to the standardly used serum samples^5,6,18^. To reduce sample impurities that could interfere with the immunoassays and achieve a concentrated IgG sample, we tested various purification methods for FVU samples, including two PGMB-based protocols and combined Amicon filtration with Melon Gel purification, referred to as AM-MG. Notably, none of these methods had been previously evaluated in the context of FVU samples. The addition of the Melon Gel purification step resulted in approximately 50% lower total IgG concentrations for the BioRad and HTRF assays, as compared to using Amicon filtration alone. Notably, both enrichment methods yielded similar concentrations when measured with AlphaLISA for total IgG and HPV16-IgG, suggesting that the difference observed in the BioRad and HTRF assays may not be attributed to IgG loss but rather to non-specific binding when residual proteins are not removed. We observed consistently high and significant Spearman correlation coefficients between HPV16-IgG concentration for both enrichment methods and serum. The additional Melon Gel filtration step might thus be valuable when IgG purity is essential.

PGMB has been widely used for IgG purification from, i.e., serum and cell culture supernatant, but has not been evaluated for FVU samples^35^. In contrast to the first two enrichment methods, the PGMB IgG enrichment methods show limited significant correlations between the IgG quantification methods. Furthermore, Spearman correlation coefficients between HPV16-IgG concentrations in serum and those obtained through PGMB-G or PGMB-CA are notably low and not statistically significant. Remarkably, the PGMB methods produced an HPV16-IgG titer for an unvaccinated FVU sample that was not detected by the other two FVU IgG processing methods or in serum. The HPV16-IgG DELFIA assay relies on time-resolved fluorescence using the lanthanide Europium (Eu^3+^)^36,37^ . Since PGMB contains magnetite (Fe_3_O_4_), and PGMB-G samples are eluted in glycine, both of which are known to interact with Eu^3+^, potential non-specific binding and contamination of exogenous lanthanides may have affected the assay’s reproducibility for samples enriched using the PGMB methods^38–41^. Therefore, the results of the PGMB for HPV16-IgG DELFIA and total IgG HTRF assays are reported as invalid^42–45^. However, it is worth noting that some correlations are observed for the PGMB enrichment when citric acid is used for elution instead of glycine, suggesting that there might be less interference with this assay for the PGMB-CA method.

### Comparison of total human IgG quantification methods

Human IgG might be important in normalizing inter- and intra-individual variation in FGT secretions collected with FVU samples. Since no assays were validated for FVU samples, our study compared three distinct total human IgG quantification methods, each with different protocols and readouts. Homogeneous assays (HTRF and AlphaLISA) offered several advantages over the BioRad ELISA-based assay, including reduced hands-on time, shorter run times, and lower variability, even though they have a narrower dynamic range^46^. These assays operate on an energy transfer mechanism between fluorophores or beads, whereas the BioRad assay relies on Luminex technology^44,47^. Importantly, all the quantification methods we employed proved to be compatible with AM and AM-MG enriched FVU samples, demonstrating significant overall correlations. As mentioned earlier, the HTRF quantification method was reported as incompatible with the PGMB enrichment methods due to non-specific binding^36^. Surprisingly, the strong correlation between the IgG quantification methods did not extend to serum samples, suggesting potential challenges or distinctions between sample types. While differences in dynamic range, matrix interactions, and distance factors between donor and acceptor may contribute to the observed variations, it is worth noting that these discrepancies were exclusive to serum samples, and the exact reasons for this distinction remain under investigation.

### Limitations

This study has certain limitations. Firstly, as this is a pilot study to evaluate protocols, the sample size is limited. Nevertheless, the obtained results provide valuable information that contributes to the evaluation of FVU as a non-invasive clinical and immunological sample. Additionally, it should be noted that HPV16-IgG levels were assessed using an in-house developed DELFIA assay, however our in-house experiments have shown strong correlations with established HPV-specific immunoassays like M9ELISA, M4ELISA, and GST-L1-MIA^18,23,48,49^. Further investigations, including prolonged storage periods at RT and − 20 °C and comparisons to unfrozen samples, could enhance our understanding of IgG stability. Moreover, it is essential to verify the outcomes of this study for samples containing naturally induced HPV16 antibodies.

## Conclusions

In conclusion, this study provides essential results that will contribute to further research exploring FVU as a valuable sample for clinical and immunological studies. Our research is the first to demonstrate that IgG in FVU samples remains stable for at least 14 days at room temperature and that the use of the UCM buffer enhances IgG detection in FVU. Furthermore, our study shows that different total IgG quantification methods are compatible with FVU samples. However, it is crucial to note that not all tested IgG enrichment methods yield scientifically accurate results with all quantification methods, and careful consideration is necessary when selecting an appropriate method for specific applications. Both the AM and AM-MG enrichment methods can be used for all analyses, with slightly superior results observed for AM-MG.

### Supplementary Information

Supplementary Information.

## Data Availability

The data presented in this study are available from the corresponding author upon reasonable request.
