# Supplementary material for: Stability, enrichment, and quantification of total and HPV16-specific IgG present in first-void urine
Source: Sci Rep. 2024 Jun 23;14:14441. doi: 10.1038/s41598-024-65257-0 (PMC11194269; doi:10.1038/s41598-024-65257-0)
Supplement: Supplementary file 1 — Supplementary Information. [file 41598_2024_65257_MOESM1_ESM.pdf]

Stability, enrichment, and quantification of total and HPV16-specific IgG present in first-void urine

Laura Téblick[1,*], Marijana Lipovac[1], Margo Bell[1], Annemie De Smet[1], Ingrid De Meester[2], Peter Delputte[3], Alex Vorsters[1]

[1] Centre for the Evaluation of Vaccination (CEV), Vaccine & Infectious Disease Institute (VAXINFECTIO), Faculty of Medicine and Health Sciences, University of Antwerp, 2610 Wilrijk-Antwerp, Belgium

[2] Laboratory of Medical Biochemistry, Faculty of Pharmaceutical, Biomedical and Veterinary Sciences, University of Antwerp, 2610 Wilrijk-Antwerp, Belgium.

[3] Laboratory for Microbiology, Parasitology and Hygiene (LMPH), Faculty of Pharmaceutical, Biomedical and Veterinary Sciences, University of Antwerp, 2610 Wilrijk-Antwerp, Belgium

Supplementary information

- Supplementary Table 1
- Supplementary Table 2
- Supplementary Table 3
- Supplementary Figure 1
- Supplementary Figure 2
- Supplementary Figure 3

Supplementary Table 1: Overview of median (IQR) total human IgG and HPV16-specific IgG concentrations in Amicon filtered FVU for different storage conditions and with different immunoassays.

	Total Human IgG			HPV16-IgG
	BioRad (µg/ml)	HTRF (µg/ml)	AlphaLISA (µg/ml)	DELFIA (IU/ml)
**No UCM**				
0 days	7.05 (2.61-13.87)	7.98 (4.00-17.73)	15.37 (3.36-19.91)	0.018 (0.004-0.071)
7 days	6.70 (2.91-15.91)	9.10 (3.89-18.43)	8.11 (3.53-21.58)	0.020 (0.006-0.070)
14 days	6.24 (2.63-14.71)	7.16 (4.29-13.54)	15.19 (3.99-19.21)	0.021 (0.005-0.112)
All	6.47 (2.20-17.82)	7.57 (4.21-15.31)	10.00 (3.39-19.44)	0.020 (0.005-0.100)
**UCM**				
0 days	9.54 (5.65-22.21)	16.97 (8.69-22.75)	14.26 (7.06-37.62)	0.024 (0.011-0.135)
7 days	8.57 (4.83-20.13)	15.78 (8.60-23.19)	10.92 (7.37-29.40)	0.022 (0.008-0.136)
14 days	9.22 (5.16-22.89)	19.08 (7.90-22.30)	11.58 ((7.07-32.89)	0.026 (0.005-0.129)
All	9.22 (5.38-27.55)	16.97 (7.54-23.19)	11.58 (6.76-35.51)	0.024 (0.009-0.154)

Supplementary Table 2: Overview of median (IQR) total human IgG and HPV16-specific IgG concentrations in FVU for different FVU enrichment methods and serum with different immunoassays. Results for TRF based methods (HTRF and DELFIA) are invalid for PGMB enrichment methods due to non-specific binding.

	Total Human IgG			HPV16-IgG
	BioRad	HTRF	AlphaLISA	DELFIA
	Concentration (µg/ml)			Concentration (IU/ml)
**FVU**				
AM	13.42 (9.81-19.60)	17.23 (9.56-31.31)	3.28 (1.74-6.23)	0.031 (0.008-0.104)
AM-MG	6.79 (3.94-9.09)	7.16 (6.06-18.20)	3.79 (0.44-4.97)	0.040 (0.006-0.086)
PGMB-G	12.91 (7.10-14.42)	~~10.49 (6.14-16.39)~~	0.89 (0.19-2.88)	~~0.206 (0.117-0.284)~~
PGMB-CA	8.19 (4.16-12.48)	~~10.98 (6.32-14.73)~~	1.16 (0.12-2.76)	~~0.203 (0.159-0.245)~~
**Serum**	3711 (2750-4216)	4029 (3401-7056)	5979 (5276-37338)	109.4 (32.6-286.9)

Supplementary Table 3: Spearman correlation coefficients between different IgG enrichment methods. Results for HTRF assay are invalid for PGMB enrichment methods due to non-specific binding.

	Total Human IgG						HPV16-IgG	
	BioRad		HTRF		AlphaLISA		DELFIA	
	Spearman correlation (95% CI)	Adjusted p-value	Spearman correlation (95% CI)	Adjusted p-value	Spearman correlation (95% CI)	Adjusted p-value	Spearman correlation (95% CI)	Adjusted p-value
**AM vs**								
AM-MG	0.89 (0.46-1.00)	0.001	0.8 (0.29-1.00)	0.005	0.72 (0.13-1.00)	0.01	0.96 (0.77-1.00)	<0.0001
PGMB-G	0.12 (-0.73-0.98)	0.78	~~0.40 (-0.40-0.96)~~	~~0.26~~	0.57 (-0.16-1.00)	0.08	~~-0.33 (-0.92-0.50)~~	~~0.36~~
PGMB-CA	0.68 (-0.01-1.00)	0.04	~~0.79 (0.22-1.00)~~	~~0.006~~	0.65 (0.00-0.96)	0.03	~~-0.009 (-0.75-0.63)~~	~~0.97~~
**AM-MG vs**								
PGMB-G	0.33 (-0.53-1.00)	0.38	~~-0.0006 (-0.70-0.69)~~	~~1~~	0.72 (0.09-1.00)	0.02	~~-0.27 (-0.86-0.46)~~	~~0.44~~
PGMB-CA	0.77 (0.13-1.00)	0.01	~~0.46 (-0.27-0.92)~~	~~0.15~~	0.86 (0.40-1.00)	0.0006	~~0.04 (-0.73-0.67)~~	~~0.92~~
**PGMB-G vs**								
PGMB-CA	0.4 (-0.47-0.98)	0.29	~~0.79 (0.33-1.00)~~	~~0.01~~	0.92 (0.63-1.00)	0.0002	~~0.52 (-0.37-0.92)~~	~~0.13~~

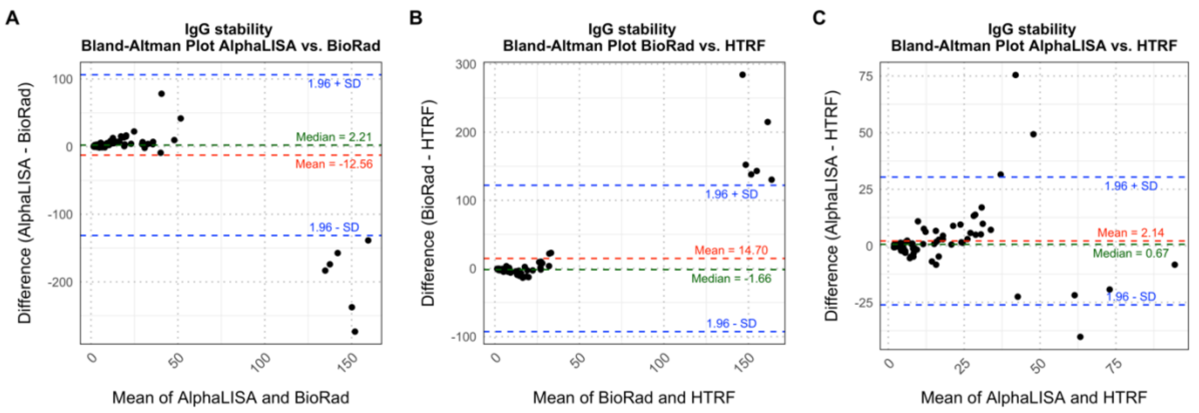

Supplementary Figure 1: Bland Altman plots for the different total human IgG quantification kits and the IgG stability cohort. The x-axis represents the mean of the measurements obtained from the two methods, while the y-axis represents the difference between the measurements. The red dashed line represents the mean of the differences, the green dashed line represents the median of the differences, and the upper and lower blue dashed lines represent the mean plus and minus 1.96 times the standard deviation (SD) of the differences, respectively.

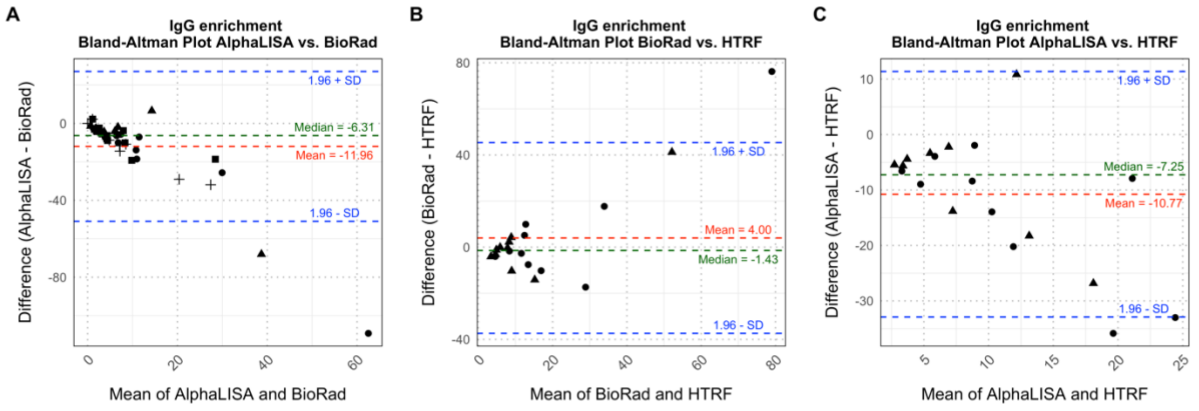

Supplementary Figure 2: Bland Altman plots for the different total human IgG quantification kits and the IgG enrichment cohort. AM results are presented as dots, AM-MG results as triangles, PGMB-G results as squares and PGMB-CA results as plus signs. Results for the HTRF assay are invalid for PGMB enrichment methods due to non-specific binding and therefore excluded for those specific analyses. The x-axis represents the mean of the measurements obtained from the two methods, while the y-axis represents the difference between the measurements. The red dashed line represents the mean of the differences, the green dashed line represents the median of the differences, and the upper and lower blue dashed lines represent the mean plus and minus 1.96 times the standard deviation (SD) of the differences, respectively.

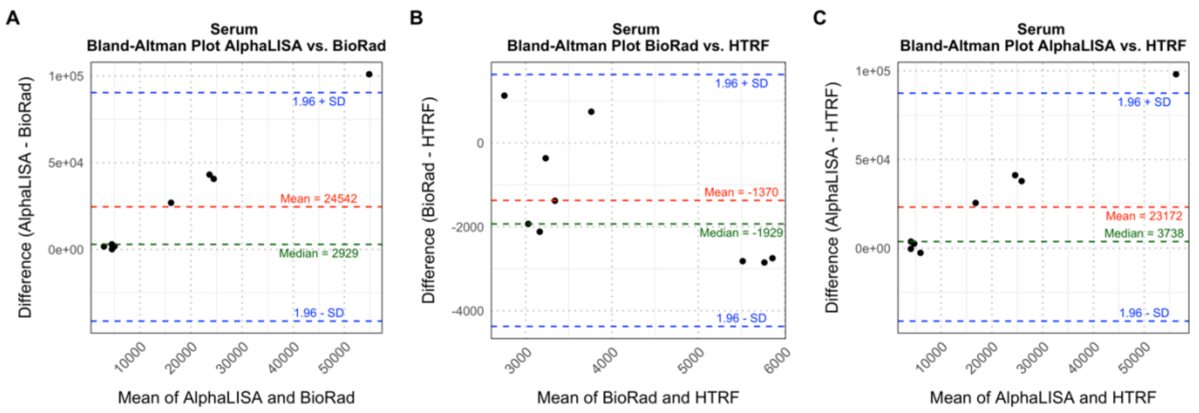

Supplementary Figure 3: Bland Altman plots for the different total human IgG quantification kits and the serum samples from the enrichment cohort. The x-axis represents the mean of the measurements obtained from the two methods, while the y-axis represents the difference between the measurements. The red dashed line represents the mean of the differences, the green dashed line represents the median of the differences, and the upper and lower blue dashed lines represent the mean plus and minus 1.96 times the standard deviation (SD) of the differences, respectively.
